# Development and initial psychometric validation of the psychological exemption zone perception scale in reverse mobility among doctoral talent

**DOI:** 10.3389/fpsyg.2026.1923326

**Published:** 2026-10-08

**Authors:** Jun Jiang, Wenfa Zhang, Peitao Du

**Affiliations:** 1 Shandong Huayu University of Technology, Dezhou, China; 2 Al-Farabi Kazakh National University, Almaty, Kazakhstan

**Keywords:** doctoral talent, higher education work setting, organizational psychology, perceived psychological exemption zone, psychological safety, reverse mobility, scale development, work-setting evaluation

## Abstract

Against the backdrop of market-oriented career environments and increasing performance pressure, some doctoral talent evaluate higher education institution/research work settings as comparatively stable, restorative, controllable, and meaningful career options. However, the existing research lacks a standardized instrument for measuring this subjective evaluation of work settings as psychologically protective environments. Building on prior constructivist grounded-theory findings and relevant organizational psychology literature, this study developed and initially validated the Psychological Exemption Zone Perception Scale (PEZPS) in the context of reverse mobility among doctoral talent. A multi-stage scale development procedure was adopted. Thirty candidate items were generated and reviewed by nine experts, resulting in a 25-item formal scale. Using data from 838 valid responses, the sample was randomly divided into an exploratory sample (*n* = 335) and a validation sample (*n* = 503). Exploratory factor analysis yielded a five-factor structure: perceived institutional shelter, perceived time sovereignty, perceived boundary controllability, perceived restorative security, and perceived meaning-belonging. The five factors explained 65.8% of the total variance, and parallel analysis supported their retention. Confirmatory factor analysis showed acceptable model fit, *χ*^2^/df = 2.324, CFI (comparative fit index) = 0.943, TLI (Tucker–Lewis index) = 0.935, RMSEA (root mean square error of approximation) = 0.051, and SRMR (standardized root mean square residual) = 0.041. PEZPS demonstrated good internal consistency, convergent validity, and discriminant validity, alongside preliminary criterion-related and incremental validity evidence. Cronbach’s *α* was 0.902 for the total scale and ranged from 0.839 to 0.881 across dimensions; CR (composite reliability) ranged from 0.834 to 0.881, AVE (average variance extracted) from 0.502 to 0.597, and the maximum HTMT (heterotrait–monotrait) value was 0.700. PEZPS total scores were significantly associated with criterion variables in theoretically expected directions and was associated with additional explained variance in reverse mobility intention beyond demographic variables and related psychological indicators. Overall, this study advances psychological exemption zone from a qualitative concept to a measurable construct and provides a preliminary tool for studying psychologically protective work-setting evaluations among doctoral talent. Further validation across different types of HEIs and broader career contexts is needed.

## 1 Introduction

In contemporary China, doctoral talent are facing increasingly complex career choices across higher education institutions (HEIs), research institutes, enterprises, internet platforms, financial institutions, high-technology industries, and other market-oriented organizations. For many doctoral students and doctoral degree holders, career choice is not determined only by income, occupational prestige, or sectoral matching. It also involves how different work settings are psychologically evaluated: whether they are perceived as risky or protective, exhausting or restorative, uncertain or predictable, and boundary-eroding or boundary-controllable. This psychological dimension is especially salient in the context of “reverse mobility,” in which some doctoral talent move, or intend to move, from enterprises and market-oriented employment imaginaries toward HEI or research work settings (Li and Horta, 2024; Tang et al., 2025; Du et al., 2026).

Prior research on doctoral career mobility has examined employment preferences, academic labor markets, sectoral transitions, institutional incentives, and human-capital allocation. These studies have clarified how different sectors attract doctoral talent under specific structural and organizational conditions (Li et al., 2024; Wang and Wang, 2024; Zhai et al., 2021). In the Chinese context, explanations of reverse mobility have often emphasized stability, institutional security, occupational prestige, welfare arrangements, academic autonomy, and work–life balance associated with HEI positions (Kallio et al., 2024; Liu et al., 2025; Tang et al., 2025). These perspectives are valuable, but they do not fully explain why some doctoral talent are willing to move away from market-oriented career paths that may offer higher income expectations, faster promotion opportunities, or stronger performance-based incentives. A more complete explanation requires attention to how individuals psychologically evaluate work settings and how these evaluations shape career intentions (Akkermans et al., 2024; De Vos et al., 2020; Sullivan and Al Ariss, 2021).

This psychological perspective was further developed in prior constructivist grounded-theory research on reverse mobility (Du et al., 2026). That study conceptualized reverse mobility as a process of setting-seeking rather than merely a sectoral transition, showing that some doctoral talent described market-oriented workplaces as a “performance competition arena” characterized by key performance indicator-driven value alienation, chronic exhaustion, anticipatory insecurity, and boundary erosion. As perceived risk accumulated, psychological safety needs became more salient, and HEI/research work settings were symbolically constructed as a “psychological exemption zone”: a perceived work setting that could reduce exposure to marketized performance risks and support recovery, predictability, meaning, and boundary controllability (Bakker et al., 2023; Edmondson and Bransby, 2023; Mellner et al., 2021). Although this prior work provided a grounded theoretical model of the psychological mechanisms underlying reverse mobility, it did not provide a standardized instrument for measuring perceived psychological exemption zone in larger samples.

This measurement gap limits further empirical research. Existing scales can assess related constructs, such as psychological safety, perceived organizational support, work autonomy, meaningful work, occupational burnout, job insecurity, and work–life balance (Edmondson and Bransby, 2023; Kurtessis et al., 2017; Shoss, 2017). However, these instruments usually focus on a single psychological experience, organizational resource, or work outcome (Blustein et al., 2023; Gagné et al., 2022; Newman et al., 2017). They do not directly measure whether individuals evaluate an entire work setting as a psychologically protective space. Without a validated scale, psychological exemption zone remains mainly a qualitative construct and cannot be systematically examined in relation to career intention, psychological needs, burnout, job insecurity, or work-setting preference.

The present study therefore develops and initially validates the Psychological Exemption Zone Perception Scale (PEZPS) in the context of reverse mobility among doctoral talent in China. Building on prior grounded-theory findings and organizational psychology literature, this study defines perceived psychological exemption zone as an individual’s subjective evaluation that a given work setting can buffer market-oriented performance risks, provide opportunities for psychological recovery, enhance career-life predictability, protect work–life boundaries, and support meaning, identity continuity, and community belonging (Du et al., 2026; Blustein et al., 2023; De Vos et al., 2020). In the present survey, the target work setting refers to the HEI/research work environment that respondents are considering, have entered, or are comparing. Thus, the concept concerns subjective work-setting evaluation rather than the objective claim that all HEIs are uniformly low-pressure or psychologically protective environments.

Based on the prior qualitative model and relevant psychological theories, PEZPS is proposed to include five dimensions. Perceived institutional shelter refers to the perception that a work setting provides relatively stable, predictable, and non-arbitrary institutional protection (Wang and Wang, 2024; Li and Horta, 2024). Perceived time sovereignty refers to perceived control over work rhythm, task progression, and autonomous time, which is conceptually aligned with autonomy-supportive work design and self-determination theory (Gagné et al., 2022; Ryan and Deci, 2020; van den Broeck et al., 2021). Perceived boundary controllability captures the perception that work–life boundaries can be maintained and protected (Kossek et al., 2012; Mellner et al., 2021; Kreiner, 2006). Perceived restorative security reflects the belief that the work setting supports recovery from long-term occupational stress and psychological depletion (Bakker et al., 2023; Sonnentag and Fritz, 2007). Perceived meaning-belonging refers to the perception that the work setting supports professional value, identity continuity, academic or teaching-related meaning, and community belonging (Blustein et al., 2023; McAnally and Hagger, 2024). Together, these dimensions translate the qualitative concept of psychological exemption zone into a measurable multidimensional construct. At a broader theoretical level, PEZPS can also be situated within sustainable-career and career-transition perspectives, which emphasize the fit between individual psychological needs and the resources and constraints of work settings across career decisions (De Vos et al., 2020; Sullivan and Al Ariss, 2021). The conceptual definitions, theoretical grounding, and representative items of the five dimensions are presented in Table 1.

**Table 1:** Theoretical dimensions of perceived psychological exemption zone.

Dimension	Conceptual meaning	Sample item
Perceived institutional shelter	An individual’s perception that a work setting can provide relatively stable, fair, predictable, and non-arbitrary institutional protection.	“This work setting would provide institutional protection for my long-term career development.”
Perceived time sovereignty	An individual’s perception of having greater control over work rhythm, task progression, and personal time.	“In this work setting, I could better organize my work rhythm.”
Perceived boundary controllability	An individual’s perception of being able to maintain relatively clear and manageable work–life boundaries.	“This work setting would help me maintain boundaries between work and life.”
Perceived restorative security	An individual’s perception that a work setting helps them recover from long-term occupational stress, physical and psychological exhaustion, and psychological risks.	“This work setting would help me recover from long-term occupational stress.”
Perceived meaning-belonging	An individual’s perception that a work setting supports personal value, professional identity, academic pursuit, and community belonging.	“This work setting would support my pursuit of meaningful professional development.”

To develop and validate PEZPS, this study first clarifies the conceptual boundaries of perceived psychological exemption zone, generates and refines an initial item pool through expert content validity review, and then examines the scale’s dimensional structure through exploratory factor analysis and parallel analysis (Boateng et al., 2018; Howard, 2016; Rogers, 2022). It further tests structural validity through confirmatory factor analysis in an independent validation sample and evaluates reliability, convergent validity, discriminant validity, criterion-related validity, and incremental validity (Goretzko et al., 2024; Henseler et al., 2015; Hu and Bentler, 1999). By transforming psychological exemption zone from a grounded theoretical concept into a quantitative measurement tool, this study provides an empirical foundation for future research on reverse mobility, psychologically protective work-setting evaluations, HEI talent attraction, and sustainable career development among doctoral talent in China.

## 2 Materials and methods

### 2.1 Participants

This study used a stratified convenience sampling strategy and targeted doctoral talent in China. Participants were eligible if they met any of the following criteria: currently pursuing a doctoral degree; holding a doctoral degree and having graduated within the past 5 years; holding a doctoral degree and currently working in an enterprise or market-oriented organization; having worked in an enterprise or market-oriented organization and subsequently entered an HEI or research institute; or considering or preparing to apply for faculty, research, or teaching-and-research positions in HEIs. A total of 884 questionnaires were collected. After removing 46 responses that failed attention checks, were incomplete, showed abnormal completion time, or exhibited obvious homogeneous response patterns, 838 valid questionnaires remained, representing 94.8% of the collected questionnaires. The sampling frame is consistent with the study’s reverse-mobility focus and with recent doctoral-career and HEI-labor-market research in China (Li and Horta, 2024; Tang et al., 2025; Wang and Wang, 2024). Data-quality screening is consistent with survey-method guidance on inattentive or insufficient-effort responding (Hong et al., 2020; Ward and Meade, 2023).

Regarding the response referent, the questionnaire instructions asked participants to judge according to their genuine understanding and expectations of faculty positions, research posts, or related work settings in HEIs. “This work setting” referred to the HEI/research work environment they were considering, had entered, or were comparing. Thus, the empirical data reflect doctoral talent’s subjective evaluations of their specific referent HEI/research work environments. In light of prior grounded theory research, teaching-oriented general undergraduate institutions, application-oriented undergraduate institutions, vocational undergraduate institutions, and other non-research-intensive HEIs constitute important applicability contexts for this construct (Du et al., 2026; Liu et al., 2025). However, this study does not claim that all participants used these types of HEIs as their only referent, nor does it describe all HEI positions as uniformly low-pressure or highly secure work settings. This treatment is not inconsistent with the prior qualitative study: the prior study provided the construct source and typical contexts, whereas the present study provides an initial scale validation within the broader context of reverse mobility among doctoral talent and evaluations of HEI/research work environments. Accordingly, the present validation spans a reverse-mobility continuum from contemplated movement to completed enterprise-to-HEI/research transitions; it should not be read as validation restricted only to completed movers.

In the final sample, 375 participants were male (44.7%) and 463 were female (55.3%). Ages ranged from 24 to 51 years, with a mean age of 32.33 years (SD = 4.45). Participants had an average of 2.00 years of enterprise experience (SD = 2.50), ranging from 0 to 13.6 years, thereby covering individuals with no enterprise experience as well as those with several years of enterprise experience. To avoid using the same data for both exploratory and confirmatory analyses, the sample was randomly split in a stratified manner by population group (career stage): approximately 40% were assigned to the exploratory sample (*n* = 335), and approximately 60% were assigned to the validation sample (*n* = 503), ensuring comparable population composition across the two subsamples. Basic sample characteristics and grouping information are shown in Table 2. The analytic split between exploratory and validation samples follows common scale-development recommendations to reduce capitalization on chance and support cross-sample structural testing (Boateng et al., 2018; Worthington and Whittaker, 2006).

**Table 2:** Basic sample characteristics.

Sample	*n*	Age M (SD)	Male	Female	Years of enterprise experience M (SD)
EFA	335	32.50 (4.57)	145 (43.3%)	190 (56.7%)	1.96 (2.46)
CFA	503	32.22 (4.37)	230 (45.7%)	273 (54.3%)	2.03 (2.52)
Total	838	32.33 (4.45)	375 (44.7%)	463 (55.3%)	2.00 (2.50)

### 2.2 Instrument development

Item generation for the PEZPS was based on three sources. First, the core categories and theoretical dimensions derived from prior constructivist grounded theory research were used (Du et al., 2026). Second, theoretical formulations in organizational psychology, vocational psychology, higher education research, and psychometrics concerning psychological safety, work autonomy, occupational burnout, meaningful work, work–life boundaries, and perceived organizational support were consulted (Bakker et al., 2023; Blustein et al., 2023; Edmondson and Bransby, 2023). Third, established item-writing conventions and Likert-type expressions from mature scales were used as references, consistent with scale-development guidelines emphasizing construct definition, item generation, expert review, and psychometric evaluation (Boateng et al., 2018; Worthington and Whittaker, 2006).

The initial item pool consisted of 30 candidate items distributed across five theoretical dimensions: perceived institutional shelter, perceived time sovereignty, perceived boundary controllability, perceived restorative security, and perceived meaning-belonging. To avoid idealizing HEIs as “pressure-free” or “competition-free” spaces, item wording consistently used qualified expressions such as “relatively stable,” “reduce concerns,” “help,” and “more likely,” thereby emphasizing subjective perception rather than objective institutional facts. All items were rated on a 5-point Likert scale, with 1 indicating “strongly disagree” and 5 indicating “strongly agree.” The formal survey items are presented in Supplementary Table S5. The cautious wording is consistent with validity-oriented recommendations that item wording should match the intended construct domain and avoid construct-irrelevant absolutism or double meanings (Boateng et al., 2018; Zapata-Ospina and García-Valencia, 2022).

### 2.3 Content validity

To examine the content validity of the initial items, nine experts with backgrounds in organizational psychology, vocational psychology, human resource management, higher education research, and psychometrics were invited to independently review the item pool. Anonymous expert background information is reported in Supplementary Table S1. Experts rated the match between each item and the construct of perceived psychological exemption zone and its assigned dimension using a three-point scale: 1 = not relevant, 2 = relevant but requires revision, and 3 = highly relevant. After rating, items were initially screened using Lawshe’s content validity ratio (CVR) (Lawshe, 1975; Wilson et al., 2012). Expert ratings, CVR values, and decision rationales for the initial items are presented in Supplementary Table S2. With nine experts, when at least eight experts rated an item as “highly relevant,” CVR = 0.78, meeting the recommended criterion. Therefore, items with CVR values of 0.78 or above were considered to have acceptable content validity (Wilson et al., 2012; Zapata-Ospina and García-Valencia, 2022).

In addition to quantitative ratings, open-ended expert comments were collected and used to revise item semantics and conceptual boundaries. For items that met the CVR criterion but were judged by experts to be insufficiently precise, revisions were made mainly in three respects. First, overly absolute wording such as “completely avoid,” “no longer anxious,” and “guarantee” was avoided so that the items better reflected realistic HEI work contexts. Second, perceived psychological exemption zone was further distinguished from non-core content such as income stability, ease of work, and general job satisfaction, ensuring that items focused on psychological perceptions of work settings, including institutional shelter, time sovereignty, boundary controllability, restorative security, and meaning-belonging. Third, for adjacent dimensions such as perceived time sovereignty and perceived boundary controllability, item wording was further clarified to reduce conceptual overlap. Deleted items and reasons for deletion are shown in Supplementary Table S3, and detailed revision trajectories for retained items are shown in Supplementary Table S4. This revision logic is consistent with content-validity evidence as a prerequisite for later construct-validity testing and with recommendations to document item-deletion rationales transparently (Boateng et al., 2018; Zapata-Ospina and García-Valencia, 2022).

The screening results of the expert content validity review are summarized in Table 3. Of the 30 initial items, 12 were retained directly because of strong expert agreement, 13 were retained after semantic revision based on expert comments, and 5 were deleted because their CVR values fell below the recommended criterion or because they involved overly absolute wording, unclear construct boundaries, or confusion with non-core content such as income security or “work being easy.” This process yielded a formal 25-item version covering five dimensions. Combining quantitative CVR thresholds with qualitative expert comments strengthens content validity by integrating numerical agreement with conceptual refinement (Lawshe, 1975; Wilson et al., 2012; Zapata-Ospina and García-Valencia, 2022).

**Table 3:** Expert content validity review results.

Decision	No. of items	CVR range	Item code
Retain	12	1.00	IS1; IS3; IS5; TS1; TS5; BC1; BC5; RS1; RS3; RS5; MB1; MB3
Retain after revision	13	0.78	IS2; IS4; TS2; TS3; TS4; BC2; BC3; BC4; RS2; RS4; MB2; MB4; MB5
Delete	5	−0.78 to 0.11	DEL1; DEL2; DEL3; DEL4; DEL5

Items retained after revision met the CVR criterion but were semantically revised based on expert comments to improve clarity, reduce absolute wording, or strengthen construct boundaries.

### 2.4 Data collection and quality control

Formal data were collected through an online questionnaire platform. The first page of the questionnaire presented the study purpose, anonymity, data use, and informed consent information; participants proceeded to the formal questionnaire only after confirming consent. To improve data quality, the questionnaire included attention-check items. Data were cleaned based on response completeness, valid value ranges, duplicate IDs, response patterns, and valid-sample flags. In the cleaned valid sample, there were no missing values for scale items, no duplicate IDs, and all item responses fell within the range of 1–5. No fully straight-lined responses were observed, and 19 cases selected the same response option for 10 or more consecutive items. These checks indicated that the cleaned data were suitable for subsequent statistical analysis. The use of attention checks, response-pattern screening, completion checks, and duplicate screening is consistent with recent best-practice recommendations for reducing careless responding and insufficient-effort responding in self-administered survey data (Hong et al., 2020; Ward and Meade, 2023; Podsakoff et al., 2003).

### 2.5 Criterion variables

To examine criterion-related validity, this study included variables such as psychological safety need, occupational burnout, job insecurity, meaningfulness of work, boundary-control need, reverse mobility intention, HEI job-search intention, and enterprise retention intention. Theoretically, perceived psychological exemption zone should be positively associated with psychological safety need, meaningfulness of work, boundary-control need, reverse mobility intention, and HEI job-search intention, and negatively associated with enterprise retention intention (Edmondson and Bransby, 2023; Blustein et al., 2023; Mellner et al., 2021). Occupational burnout and job insecurity reflect the stress, depletion, and uncertainty that individuals face in market-oriented work settings (Bakker et al., 2023; Shoss, 2017; Sverke et al., 2019). Theoretically, when individuals accumulate higher psychological costs in market-oriented settings, they are more likely to actively seek alternative protective settings; therefore, perceived psychological exemption zone was expected to be significantly and positively related to both variables.

The criterion variables in this study were used primarily as external reference indicators to provide an initial test of the criterion-related and incremental validity of PEZPS; they were not the core measurement instruments of this study. To control questionnaire length and respondent burden, these variables were operationalized using brief indicators, each rated from 1 to 5. For criterion variables operationalized with single items, Cronbach’s *α* was not calculated; the related limitation is addressed in Section 4. Operational definitions and theoretical uses of the criterion variables are shown in Table 4. The use of brief single-item indicators can be defensible for preliminary criterion reference or model-control purposes when questionnaire burden is high, although such indicators require careful interpretation and cannot provide internal consistency estimates (Fisher et al., 2016; Allen et al., 2022).

**Table 4:** Operationalization of criterion variables.

Variable	Operationalization and theoretical use	Scoring/reliability note
Psychological safety need	Reflects individuals’ need for a stable, low-risk, and psychologically protective environment in career choice; expected to be positively correlated with PEZPS.	1–5 points; brief criterion indicator, *α* not applicable
Occupational burnout	Reflects individuals’ subjective experience of high-pressure work and persistent depletion; as a stress-driven criterion, expected to be positively correlated with PEZPS.	1–5 points; brief criterion indicator, *α* not applicable
Job insecurity	Reflects concerns about career continuity, job security, and future uncertainty; as a stress-driven criterion, expected to be positively correlated with PEZPS.	1–5 points; brief criterion indicator, *α* not applicable
Meaningfulness of work	Reflects individuals’ pursuit of professional development, value realization, and work meaning; expected to be positively correlated with PEZPS.	1–5 points; brief criterion indicator, *α* not applicable
Boundary-control need	Reflects individuals’ need for work–life boundaries, personal time, and order in life; expected to be positively correlated with PEZPS.	1–5 points; brief criterion indicator, *α* not applicable
Reverse mobility intention	Reflects individuals’ tendency to shift from enterprises or market-oriented employment imaginaries toward HEI/research positions; used as the primary criterion outcome variable.	1–5 points; brief criterion indicator, *α* not applicable
HEI job-search intention	Reflects individuals’ intention to apply for faculty, research, or related positions in HEIs in the future; expected to be positively correlated with PEZPS.	1–5 points; brief criterion indicator, *α* not applicable
Enterprise retention intention	Reflects individuals’ tendency to remain in enterprises or market-oriented organizations; expected to be negatively correlated with PEZPS.	1–5 points; brief criterion indicator, *α* not applicable

Because the criterion variables were brief single-item indicators, their scoring methods and theoretical uses are summarized in Table 4, and the full item wording is reported in Supplementary Table S12. Single-item indicators are suitable for preliminary criterion references and model-control contexts, such as the present study’s need to control questionnaire length and respondent burden, but their reliability cannot be estimated using internal consistency coefficients. This limitation is discussed below. Recent methodological discussions support cautious and construct-specific use of single-item measures, particularly when a construct is concrete, respondent burden is a major concern, or the indicator is used as an external criterion rather than a full latent scale (Fisher et al., 2016; Allen et al., 2022).

### 2.6 Statistical analysis

Data analyses followed common procedures in scale development and psychometric validation research. First, item descriptive statistics and item analyses were conducted, including means, standard deviations, skewness, kurtosis, corrected item-total correlations, and critical ratios between high- and low-score groups. Second, exploratory factor analysis was conducted in the exploratory sample. Because the dimensions of perceived psychological exemption zone are theoretically interrelated, principal axis factoring was used for factor extraction, and Promax oblique rotation (Kappa = 4) was used to report the rotated pattern matrix, consistent with the theoretical assumption of correlated dimensions and the subsequent correlated first-order five-factor confirmatory model (Howard, 2016; Rogers, 2022; Worthington and Whittaker, 2006). The number of factors retained was determined by integrating eigenvalues, scree plot inspection, parallel analysis (Horn, 1965; 500 random simulations), and theoretical interpretability. These analyses were conducted using the Python factor_analyzer package with a fixed random seed. Because different statistical software packages may differ slightly in implementations of oblique rotation and loading standardization, the item-level loadings reported in Supplementary Table S6 are based on the output of the same Python script and parameter settings used in this study. The analysis script may be obtained from the corresponding author upon reasonable request for research purposes, subject to appropriate ethical and data-use considerations. Third, confirmatory factor analysis was conducted in an independent validation sample using the Python semopy package with maximum likelihood (ML) estimation. Given the five response categories and the absence of severe item skewness, ML was used as an approximate continuous-data estimator for this initial validation; the absence of an ordinal-estimator sensitivity analysis is treated as a limitation. Model-fit indices including *χ*^2^/df, CFI, TLI, RMSEA, and SRMR were reported (Goretzko et al., 2024; Hu and Bentler, 1999; Igolkina and Meshcheryakov, 2020). Fourth, Cronbach’s *α*, composite reliability (CR), average variance extracted (AVE), and the heterotrait–monotrait (HTMT) ratio were used to test internal consistency, convergent validity, and discriminant validity (Fornell and Larcker, 1981; Henseler et al., 2015). Fifth, correlation analyses and hierarchical regressions were used to examine preliminary criterion-related validity evidence, and hierarchical regressions were further used to examine the incremental association of PEZPS with reverse mobility intention after controlling for related psychological needs and stress indicators. In addition, Harman’s single-factor test was used as a preliminary diagnostic of common method variance, and confirmatory factor analysis was used to compare the correlated first-order five-factor model with a second-order factor model (Podsakoff et al., 2003). Model-fit criteria followed Hu and Bentler (1999), while recent critiques of fixed cutoffs were also considered when interpreting global fit indices as reference guidelines rather than absolute rules (Goretzko et al., 2024; Groskurth et al., 2024; McNeish and Wolf, 2023). PEZPS scores across career-stage groups were presented descriptively only. To preliminarily examine the applicability of the five-factor structure across career-stage groups, correlated first-order five-factor models were fitted separately within each group.

## 3 Results

### 3.1 Item analysis

Descriptive statistics in the exploratory sample showed that the means of the 25 formal items ranged from 2.92 to 3.28 and standard deviations ranged from 1.13 to 1.28. Skewness ranged from −0.242 to 0.147, and kurtosis ranged from −1.078 to −0.714, all within acceptable ranges, indicating that the item distributions were not severely skewed. Corrected item-total correlations ranged from 0.392 to 0.576. Critical-ratio tests between high- and low-score groups showed significant differences for all items, with *t* values ranging from 7.981 to 16.001, all *p* < 0.001. The item-analysis results supported retaining all 25 items for factor analysis.

### 3.2 Exploratory factor analysis and parallel analysis

The KMO value in the exploratory sample was 0.902, and Bartlett’s test of sphericity was significant, *χ*^2^ = 4416.8, df = 300, *p* < 0.001, indicating that the correlation matrix was suitable for factor analysis. Exploratory factor analysis showed that the five extracted factors cumulatively explained 65.8% of the total variance (whereas the sum of the first five initial eigenvalues accounted for 66.4% of item variance). Primary loadings on the theoretically assigned factors ranged from 0.737 to 0.895, communalities ranged from 0.549 to 0.820, and the maximum cross-loading was 0.142. No severe cross-loadings that would alter item dimensional assignment were observed. Complete item loading results are reported in Supplementary Table S6.

Parallel analysis further supported the five-factor structure. The comparison between empirical eigenvalues and random simulated mean eigenvalues is shown in Table 5, and the scree plot is shown in Figure 1. The first five empirical eigenvalues were 7.783, 3.343, 2.219, 1.719, and 1.542, each exceeding the corresponding random simulated mean eigenvalue. The sixth empirical eigenvalue was 0.886, which was lower than the random simulated mean eigenvalue of 1.238. Therefore, consistent with the theoretical five-dimensional framework, this study retained five factors: perceived institutional shelter, perceived time sovereignty, perceived boundary controllability, perceived restorative security, and perceived meaning-belonging.

**Table 5:** Parallel analysis: comparison of empirical and random simulated eigenvalues.

Factor	Empirical eigenvalue	Random simulated mean eigenvalue	Decision
1	7.783	1.535	Retain
2	3.343	1.450	Retain
3	2.219	1.387	Retain
4	1.719	1.332	Retain
5	1.542	1.284	Retain
6	0.886	1.238	Do not retain
7	0.700	1.196	Do not retain
8	0.592	1.156	Do not retain

The criterion for factor retention was that the empirical eigenvalue exceeded the random simulated mean eigenvalue.

**Figure 1:**
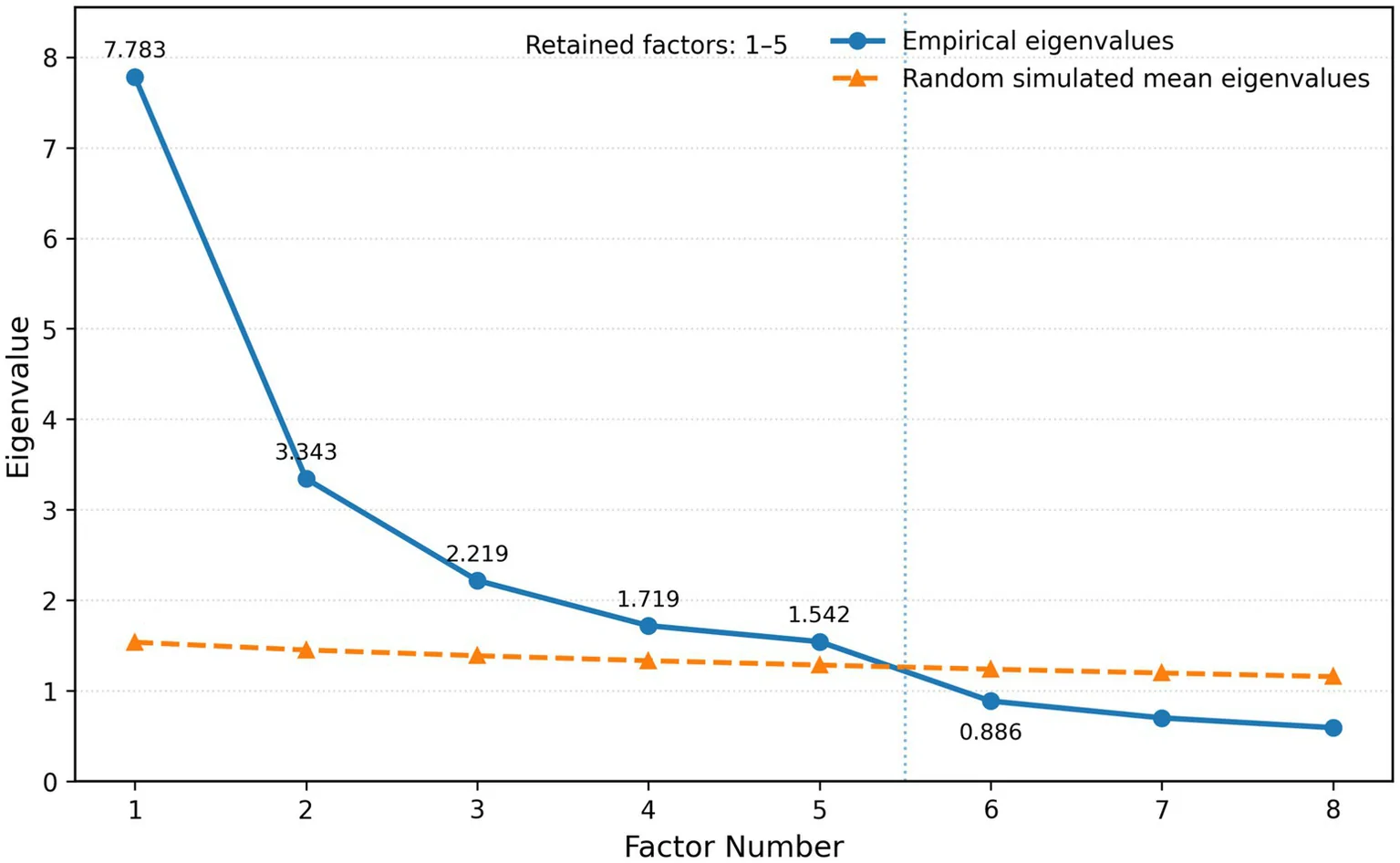
Parallel analysis scree plot.

### 3.3 Confirmatory factor analysis

The five-factor structure derived from exploratory factor analysis was tested in the validation sample. Perceived institutional shelter, perceived time sovereignty, perceived boundary controllability, perceived restorative security, and perceived meaning-belonging were specified as five correlated first-order latent variables, each measured by five items. Confirmatory factor analysis indicated that the correlated first-order five-factor model fit the data well: *χ*^2^/df = 2.324, CFI = 0.943, TLI = 0.935, RMSEA = 0.051, SRMR = 0.041. Model-fit indices are summarized in Table 6, and the five-factor measurement model is illustrated in Figure 2. Standardized factor loadings for all items are reported in Supplementary Table S7.

**Table 6:** Confirmatory factor analysis model-fit indices.

Index	Value	Target range	Decision
*χ*^2^/df	2.324	<3	Met
CFI	0.943	≥ 0.90	Met
TLI	0.935	≥ 0.90	Met
RMSEA	0.051	< 0.08	Met
SRMR	0.041	< 0.08	Met

**Figure 2:**
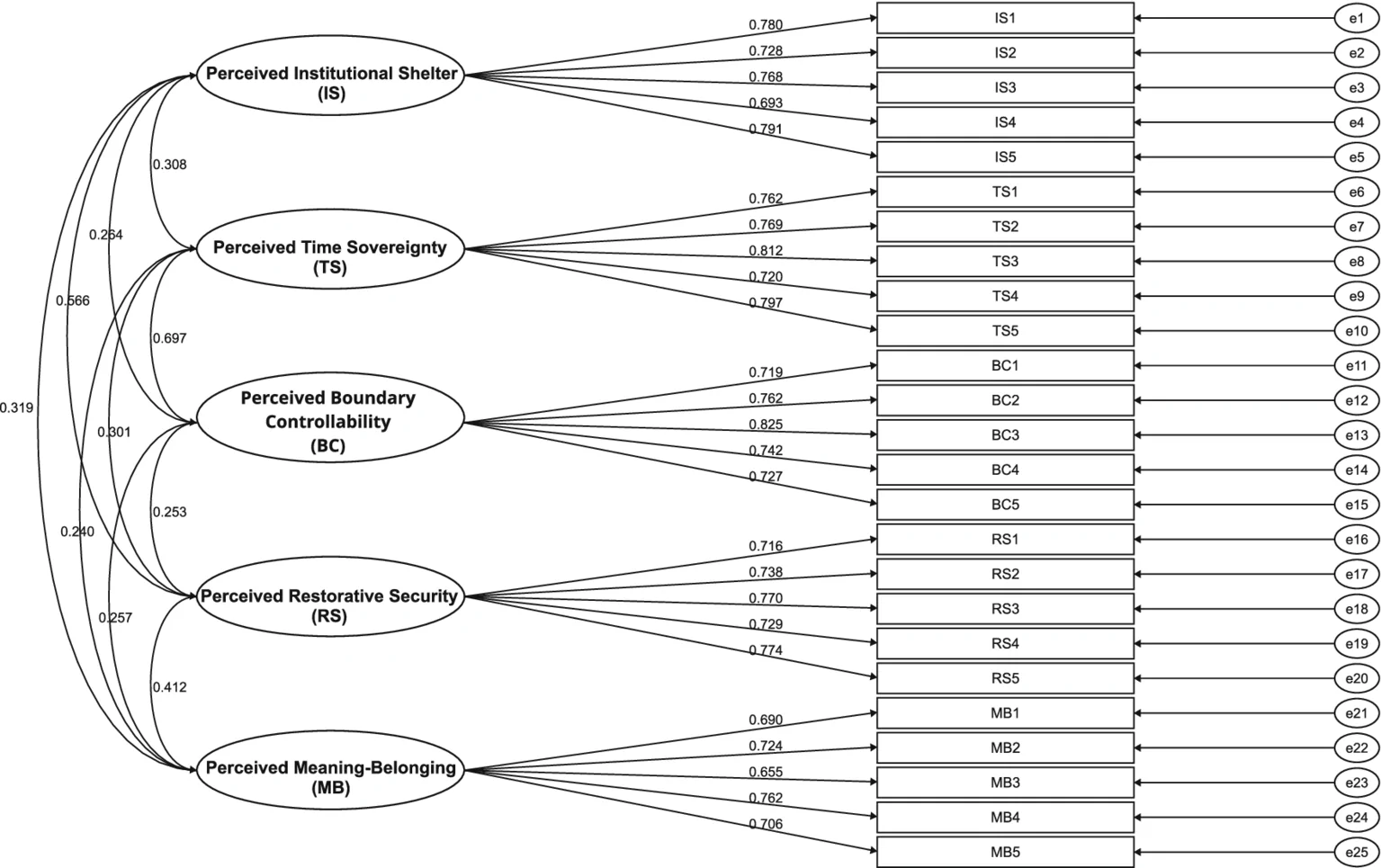
Schematic diagram of the PEZPS five-factor measurement model. Rectangles represent observed variables, ellipses represent latent variables, and path coefficients are standardized factor loadings. Correlations among dimensions are indicated by bidirectional arrows, with correlation coefficients labeled above the curves on the left. The corresponding correlation coefficients are also reported in Supplementary Table S9.

To further examine the rationale for aggregation and comparison using PEZPS total scores, an additional second-order factor model was tested in the validation sample. In this model, perceived institutional shelter, perceived time sovereignty, perceived boundary controllability, perceived restorative security, and perceived meaning-belonging were specified as first-order indicators of a single higher-order factor, perceived psychological exemption zone. The second-order model also achieved acceptable fit: *χ*^2^/df = 2.797, CFI = 0.921, TLI = 0.913, RMSEA = 0.060. Its fit was slightly lower than that of the correlated first-order five-factor model, as generally expected for higher-order models. This result suggests that the five dimensions are relatively distinct but can also be subsumed under a higher-order perceived psychological exemption zone construct, providing preliminary structural support for cautious use of PEZPS total scores while retaining the five subscales as the primary multidimensional interpretation.

### 3.4 Reliability, convergent validity, and discriminant validity

Reliability analysis showed that Cronbach’s *α* was 0.902 for the total PEZPS and ranged from 0.839 to 0.881 across the five dimensions, all reaching acceptable levels. Regarding convergent validity, CR ranged from 0.834 to 0.881, all above 0.70, and AVE ranged from 0.502 to 0.597, reaching or exceeding 0.50. These results indicate that each latent variable explained a substantial proportion of variance in its items. The reliability, convergent validity, and discriminant validity results are summarized in Table 7.

**Table 7:** Summary of reliability, convergent validity, and discriminant validity.

Dimension	Cronbach’s *α*	CR	AVE	Maximum HTMT	Decision
Perceived institutional shelter	0.865	0.867	0.567	0.572	Met
Perceived time sovereignty	0.881	0.881	0.597	0.700	Met
Perceived boundary controllability	0.870	0.869	0.572	0.700	Met
Perceived restorative security	0.871	0.862	0.556	0.572	Met
Perceived meaning-belonging	0.839	0.834	0.502	0.413	Met

For discriminant validity, HTMT ratios ranged from 0.240 to 0.700, all below the 0.85 criterion, indicating that although the five dimensions were correlated, they remained sufficiently distinct. The Fornell–Larcker criterion was also used to further assess discriminant validity. Results showed that the square roots of AVE for all five dimensions (0.709–0.773) exceeded their correlations with other dimensions. The highest inter-dimensional correlation occurred between perceived time sovereignty and perceived boundary controllability (*r* = 0.697), which was still lower than the square roots of their AVE values. The complete HTMT and Fornell–Larcker matrices are provided in Supplementary Tables S8, S9.

### 3.5 Criterion-related validity

Correlation results are shown in Table 8. PEZPS total scores were significantly and positively correlated with psychological safety need, occupational burnout, job insecurity, meaningfulness of work, boundary-control need, reverse mobility intention, and HEI job-search intention, and significantly and negatively correlated with enterprise retention intention. The directions of these correlations were consistent with theoretical expectations. Correlations among criterion variables were generally interpretable and did not show abnormally high values. For interpretive clarity, this study classified the criterion variables into three groups: convergent variables (psychological safety need, meaningfulness of work, and boundary-control need), concurrent outcome variables (reverse mobility intention, HEI job-search intention, and enterprise retention intention), and stress-driven variables (occupational burnout and job insecurity). The positive correlations between occupational burnout/job insecurity and PEZPS are not a discriminant-validity problem; rather, they reflect the theoretical expectation that stress and uncertainty in market-oriented work settings may drive individuals to construct HEIs and similar settings as psychologically protective spaces.

**Table 8:** Descriptive statistics and correlation matrix for PEZPS total score and criterion variables.

Variable	M	SD	A	B	C	D	E	F	G	H	I
A	3.091	0.648	—								
B	2.967	1.185	0.640**	—							
C	2.961	1.130	0.430**	0.298**	—						
D	3.036	1.113	0.454**	0.333**	0.144**	—					
E	3.088	1.172	0.593**	0.381**	0.259**	0.271**	—				
F	3.000	1.148	0.604**	0.374**	0.293**	0.279**	0.310**	—			
G	3.026	1.112	0.584**	0.364**	0.237**	0.276**	0.355**	0.347**	—		
H	2.927	1.090	0.510**	0.301**	0.225**	0.216**	0.307**	0.270**	0.307**	—	
I	2.995	1.125	−0.467**	−0.257**	−0.221**	−0.201**	−0.257**	−0.313**	−0.280**	−0.242**	—

*N* = 838. Pearson correlation coefficients are reported with two-tailed tests. **p < 0.01. The upper triangle is left blank to avoid duplicate reporting. A = PEZPS total score; B = psychological safety need; C = occupational burnout; D = job insecurity; E = meaningfulness of work; F = boundary-control need; G = reverse mobility intention; H = HEI job-search intention; I = enterprise retention intention.

To examine the incremental association between perceived psychological exemption zone and reverse mobility intention, reverse mobility intention was first regressed on PEZPS total scores using hierarchical regression. After controlling for gender, age, disciplinary background, career stage, and years of enterprise experience, adding PEZPS total score increased model explanatory power from *R*^2^ = 0.022 to *R*^2^ = 0.349 (Δ*R*^2^ = 0.327), with a standardized regression coefficient of *β* = 0.579, *p* < 0.001. This result indicates that perceived psychological exemption zone was associated with additional explained variance in reverse mobility intention beyond demographic and career-background variables; given the cross-sectional design, this association should not be interpreted as evidence of prediction over time or causal influence.

On this basis, to further examine the incremental validity of PEZPS relative to related psychological needs and stress indicators, a three-step hierarchical regression was conducted with reverse mobility intention as the dependent variable. Model 1 included the control variables described above. Model 2 added psychological safety need, occupational burnout, job insecurity, meaningfulness of work, and boundary-control need. Model 3 further added PEZPS total score (see Table 9). Results showed that adding PEZPS to Model 2 increased explanatory power from *R*^2^ = 0.249 to *R*^2^ = 0.350, Δ*R*^2^ = 0.101, Δ*F*(1, 820) = 126.940, *p* < 0.001; the standardized regression coefficient for PEZPS total score was *β* = 0.582, *p* < 0.001. It should be noted that the similarity between the explanatory power of Model 3 (*R*^2^ = 0.350) and the above regression including only control variables and PEZPS (*R*^2^ = 0.349) is coincidental, as the two models include different predictors. The former examines the incremental association of PEZPS relative to related psychological constructs, whereas the latter examines its association with reverse mobility intention after adjustment for demographic and career-background variables. These findings indicate that PEZPS does not merely duplicate psychological safety need, occupational burnout, job insecurity, meaningfulness of work, or boundary-control need, but instead accounts for additional variance in reverse mobility intention.

**Table 9:** Hierarchical regression of reverse mobility intention on PEZPS.

Model	Main variables entered	*R* ^2^	Adjusted *R*^2^	Δ*R*^2^	Δ*F*	PEZPS *β*
Model 1	Gender, age, disciplinary background, career stage, and years of enterprise experience	0.022	0.009	0.022	—	—
Model 2	Model 1 + psychological safety need, occupational burnout, job insecurity, meaningfulness of work, and boundary-control need	0.249	0.235	0.227	49.739***	—
Model 3	Model 2 + PEZPS total score	0.350	0.337	0.101	126.940***	0.582***

*N* = 838. ΔF refers to the test of change in explanatory power relative to the preceding model. ***p < 0.001. PEZPS *β* is the standardized regression coefficient of PEZPS total score in Model 3.

To avoid overinterpreting cross-group differences, PEZPS means across doctoral talent groups at different career stages are presented only descriptively in Supplementary Table S10. The table is intended to show the distribution of perceived psychological exemption zone across career-stage groups rather than to support strong inferential comparisons of group means. The core inferences of this study, including the validity of the five-factor structure, reliability and validity evidence, preliminary criterion-related validity evidence, and the regression-based association between perceived psychological exemption zone and reverse mobility intention, are based on the total sample or the independent validation sample and do not depend on mean comparisons among career-stage groups. Therefore, the group mean differences in Supplementary Table S10 should be interpreted only as exploratory descriptive results, not as inferential evidence for the study’s core conclusions.

To further examine the applicability of the five-factor structure across career-stage groups, correlated first-order five-factor models were fitted separately in the five doctoral talent groups. Results showed that model fit was acceptable in all groups (fit indices are reported in Supplementary Table S11), with CFI = 0.928–0.947 and RMSEA = 0.050–0.061. These results indicate that the five-factor structure of perceived institutional shelter, perceived time sovereignty, perceived boundary controllability, perceived restorative security, and perceived meaning-belonging showed good structural consistency across career-stage groups. It should be noted, however, that these results provide only preliminary support for configural consistency across groups and do not constitute strict tests of loading invariance or intercept invariance.

### 3.6 Common method bias test

As the predictor and criterion variables in this study were collected from the same self-report source, common method bias may be present. Harman’s single-factor test was therefore used as a preliminary assessment. First, all 25 PEZPS items were entered into an unrotated exploratory factor analysis, and the first common factor explained 30.08% of the total variance. Second, the 25 PEZPS items and all criterion variables were entered together into an unrotated exploratory factor analysis, and the first common factor explained 30.34% of the total variance. Both values were below the 50% reference threshold proposed by Podsakoff et al. (2003), indicating that no single factor accounted for a dominant share of the variance. It should be noted that Harman’s single-factor test provides only preliminary evidence and cannot completely rule out the possibility of common method bias. Therefore, procedural controls, including anonymous responding, informed consent, attention-check items, exclusion of invalid questionnaires, and control of variable order, were also implemented. Taken together, these results indicate that no single dominant method factor was detected; however, because predictor and criterion variables were collected from the same self-report source within a single survey, common method variance cannot be fully ruled out, and this remains a limitation of the present design.

## 4 Discussion

### 4.1 Main findings

Building on the concept of “psychological exemption zone” proposed in prior grounded theory research, this study developed and initially validated the PEZPS in the context of reverse mobility among doctoral talent. Without exceeding the formal survey framework, the study also discussed its theoretical applicability in teaching-oriented general undergraduate institutions, application-oriented undergraduate institutions, vocational undergraduate institutions, and other non-research-intensive HEIs. Results showed that PEZPS consists of five dimensions—perceived institutional shelter, perceived time sovereignty, perceived boundary controllability, perceived restorative security, and perceived meaning-belonging—comprising 25 items. Exploratory factor analysis, parallel analysis, and confirmatory factor analysis jointly supported the five-factor structure. This indicates that perceived psychological exemption zone is neither a single expectation of stability nor a simple imagination that work is easy, but rather an integrated work-setting evaluation encompassing institutional, temporal, boundary-related, restorative, and meaning-related aspects.

Parallel analysis showed that the first five empirical eigenvalues were all higher than the random simulated mean eigenvalues, whereas the sixth empirical eigenvalue was lower than the random simulated mean. This suggests that the five-factor structure is supported not only theoretically but also by exploratory statistical evidence, providing a stronger empirical basis for retaining the five dimensions of perceived institutional shelter, perceived time sovereignty, perceived boundary controllability, perceived restorative security, and perceived meaning-belonging.

Reliability and validity results further indicated that PEZPS has good psychometric quality. Cronbach’s *α* values for the total scale and all dimensions reached acceptable levels, CR and AVE supported convergent validity, and HTMT supported discriminant validity. Notably, the AVE for the perceived meaning-belonging dimension was 0.502. Although close to the 0.50 threshold, it still met the commonly used criterion, indicating that this dimension can be retained and further examined in future research.

Criterion-related results showed that PEZPS was significantly and positively associated with psychological safety need, meaningfulness of work, boundary-control need, reverse mobility intention, and HEI job-search intention, and significantly and negatively associated with enterprise retention intention. This suggests that perceived psychological exemption zone is not merely a general preference for HEIs; rather, it is closely related to doctoral talent’s career needs for safety, recovery, boundaries, and meaning. At the same time, PEZPS was positively correlated with occupational burnout and job insecurity, suggesting that higher perceived career pressure and uncertainty were associated with a stronger tendency to evaluate HEIs or similar work settings as psychologically protective spaces; given the cross-sectional design, this association should not be interpreted as evidence that burnout or job insecurity cause doctoral talent to construct HEIs as psychological exemption zones. More importantly, incremental validity regression showed that PEZPS still accounted for additional variance in reverse mobility intention after related psychological needs and stress indicators were controlled, supporting its incremental association with reverse mobility intention as a distinct construct rather than establishing a causal or temporally predictive relationship.

The descriptive group means in Supplementary Table S10 showed that participants who had moved from enterprises into HEIs had relatively higher PEZPS total scores. This finding suggests that perceived psychological exemption zone may be related to actual career transition experience, but it remains cross-sectional descriptive evidence and cannot be directly interpreted as showing that career transition causes higher perceived psychological exemption zone.

### 4.2 Alignment with prior research and scale use scope

The present study and the prior qualitative study are progressive rather than conflicting in scope. The prior study proposed a process model of “performance competition arena–psychological exemption zone” through interviews and found that teaching-oriented general undergraduate institutions, application-oriented undergraduate institutions, vocational undergraduate institutions, and other non-research-intensive HEIs were more readily imagined by participants as restorative, predictable, and boundary-controllable target settings. This study does not directly equate that qualitative contextual finding with the sole boundary of empirical scale validation. Instead, it translates the finding into the construct source, theoretical interpretive focus, and priority contexts for subsequent validation of PEZPS.

Accordingly, the current evidence for PEZPS should be understood as preliminary psychometric evidence in the context of reverse mobility among doctoral talent and evaluation of HEI/research work environments. If future research intends to use the scale specifically to compare teaching-oriented general undergraduate institutions, application-oriented undergraduate institutions, vocational undergraduate institutions, or other non-research-intensive HEIs, the questionnaire instructions should explicitly define the referent HEI type, and stratified sampling, CFA replication, multi-group measurement invariance testing, and predictive validity tests should be conducted. This approach frames “applicability to specific HEI types” as a subsequent validation issue concerning scale-use scope, rather than as a challenge to the validity of the present data or its consistency with prior research.

### 4.3 Theoretical contributions

First, this study advances psychological exemption zone from a qualitative theoretical concept to a measurable psychological construct. Prior grounded theory research revealed how doctoral talent construct HEIs or related work environments as psychologically protective settings in the context of market-oriented performance risks. The present study further translates this processual concept into a standardized measurement tool with a clear dimensional structure and statistical evidence.

Second, this study extends the level of psychological safety research. Traditional psychological safety research has largely focused on interpersonal risk taking within teams (Edmondson, 1999; Edmondson and Lei, 2014), whereas perceived psychological exemption zone emphasizes individuals’ evaluations of the safety, restorability, and sustainability of an entire occupational field. Doctoral talent are not merely searching for a specific job; they are searching across different institutional arrangements, evaluation logics, temporal structures, and meaning systems for environments that can better sustain psychological safety and career development.

Third, this study enriches research on reverse mobility among doctoral talent. Existing explanations have mostly emphasized external conditions such as HEI stability, status prestige, and institutional security. This study instead reveals the internal mechanisms of reverse mobility from the perspectives of psychological risk perception, occupational burnout, boundary erosion, and meaning restoration. The incremental analyses further suggest that perceived psychological exemption zone is not a simple substitute for occupational burnout, meaningfulness of work, or boundary-control need, but an integrated work-setting evaluation construct that accounts for additional variance in reverse mobility intention beyond these variables. Fourth, this study provides a new tool for interdisciplinary research across higher education management, human resource management, and organizational psychology. It can be used to analyze relationships among HEI attractiveness, psychological risks in market-oriented organizations, and career choices of doctoral talent. At the same time, this study explicitly treats applicability across different types of HEIs as an issue for subsequent validation, helping to avoid overextending typical-context findings from prior qualitative research into the sole applicability boundary of the current scale.

### 4.4 Practical implications

For HEIs, the PEZPS can help assess their psychological attractiveness to doctoral talent. In talent recruitment, HEIs should not emphasize only salary, job titles, and research conditions; they should also attend to institutional transparency, evaluation cycles, work rhythm, boundary protection, and the construction of academic communities.

For enterprises, PEZPS can reveal why highly educated talent leave market-oriented organizations. If doctoral talent commonly perceive HEIs as more restorative and boundary-controllable settings, this may indicate problems in enterprises such as excessive performance orientation, insufficient recovery resources, impaired psychological safety, and weakened work meaning. Enterprises seeking to retain doctoral talent need systematic improvements in performance management, recovery resources, boundary protection, and long-term development mechanisms.

For doctoral talent, the scale can be used for career reflection and career counseling. Higher perceived psychological exemption zone may indicate stronger needs for safety, recovery, and boundaries, but it may also include idealized expectations of HEI work. Therefore, individuals making career decisions should identify their real psychological needs while also gaining a deeper understanding of the actual evaluation systems, workloads, and development pathways of target HEI positions.

### 4.5 Limitations and future research

First, the sample in this study consisted mainly of doctoral talent in China. Whether the findings apply to other countries, higher education systems, and labor market contexts requires further validation. It should be emphasized that the sample included both individuals with enterprise experience who had moved into HEIs or research institutes and senior doctoral students, recent doctoral graduates, and individuals with HEI job-search intentions. Therefore, “reverse mobility” in this study includes both actual completed movement from market-oriented organizations toward HEIs or research institutes and pre-mobility intentions involving a shift from market-oriented employment imaginaries toward HEI/research work environments. It should further be noted that this study did not conduct formal sensitivity or subgroup comparisons (e.g., participants with versus without enterprise experience, or actual movers versus intention-based participants); such comparisons would help clarify whether the factor structure and criterion associations of PEZPS are stable across these subgroups, and this is left to future research.

Second, the use scope of PEZPS should be understood in relation to the present survey framework. In the formal survey, “this work setting” was used as the referential expression, and participants responded according to the HEI/research work environment they were considering, had entered, or were comparing. They were not required to use any specific type of HEI as the only referent. Therefore, the evidence obtained in this study primarily supports PEZPS as a preliminary measurement tool in the context of reverse mobility among doctoral talent and evaluation of HEI/research work settings. Teaching-oriented, application-oriented, vocational undergraduate, and other non-research-intensive HEIs are important theoretical contexts identified in prior qualitative research, but the present study did not conduct dedicated stratified sampling or multi-group measurement invariance testing across HEI types. In other words, applicability across different types of HEIs is a matter of scale-use scope and subsequent cross-context validation, rather than a negation of the validity of the present data, the basic evidence for structural validity, or consistency with prior research. Future studies should explicitly define the referent HEI type and further compare the structural stability, measurement invariance, and predictive validity of PEZPS across research universities, teaching-oriented HEIs, application-oriented undergraduate institutions, vocational undergraduate institutions, and research institutes.

Third, this study used a cross-sectional design and therefore cannot infer causal relationships between perceived psychological exemption zone and reverse mobility intention. Future research may adopt longitudinal designs to examine how perceived psychological exemption zone changes across career stages, job-search experiences, and entry experiences, and to further test its predictive validity for actual career transition behavior. Fourth, some criterion variables in this study were brief single-item indicators. They are suitable for preliminary criterion reference and model-control purposes, but they cannot replace mature multi-item scales. Future studies can use established scales for occupational burnout, meaningful work, work–life boundaries, and job-search intention to further verify the present conclusions. Fifth, confirmatory factor analysis in this study was conducted using maximum likelihood estimation, which treats the five-point Likert items as continuous; this is a common but not the only defensible approach for ordinal data, and a sensitivity analysis using an estimator designed for ordinal indicators, such as WLSMV based on polychoric correlations, was not conducted. Replicating the five-factor structure with such an estimator would strengthen the evidence for structural validity. Sixth, although a second-order factor model achieved acceptable fit and provided some support for aggregating the five dimensions into a PEZPS total score, this study did not report second-order factor loadings in detail or compute a hierarchical reliability estimate (e.g., hierarchical omega) for the general factor. Justification for treating PEZPS as a unidimensional total score should therefore be regarded as preliminary, and researchers who wish to emphasize the general factor specifically, or who find limited support for it in their own data, may prefer to analyze and report the five subscale scores separately.

Seventh, although this study preliminarily examined the applicability of the five-factor structure across doctoral talent groups at different career stages and found that five-factor models fitted separately within each group achieved acceptable fit, thereby providing preliminary evidence for configural consistency, strict multi-group measurement invariance tests have not yet been conducted. This is partly due to the relatively small sample sizes of some groups, with the smallest group containing 104 participants. In addition, postdoctoral researchers and early-career HEI faculty were combined into one analytic group in this study. Although these participants are broadly located at early stages of academic careers and some early-career faculty transition from postdoctoral positions, differences in job nature, institutional status, and career stability may still exist. Future studies with larger and more balanced samples should examine these two groups separately. In particular, loading invariance and intercept invariance have not been tested. Because intercept invariance is generally regarded as an important prerequisite for comparing latent means across groups, PEZPS means across career-stage groups are presented only descriptively in this study and are not interpreted as strong inferential evidence. Future research should systematically test measurement invariance of PEZPS across gender, disciplinary background, career stage, organizational experience, and target HEI type under conditions of larger and more balanced group sample sizes.

## 5 Conclusion

This study developed and initially validated the PEZPS in the context of reverse mobility among doctoral talent. Based on prior grounded theory and organizational psychology theory, the scale measures doctoral talent’s subjective evaluations of the HEI/research work environments they are considering, have entered, or are comparing in terms of institutional shelter, time sovereignty, boundary controllability, restorative security, and meaning-belonging. In combination with prior qualitative research, PEZPS can provide a tool foundation for subsequent analyses of psychologically protective work-setting evaluations in relatively low short-cycle research-performance-pressure contexts, such as teaching-oriented, application-oriented, vocational undergraduate, and other non-research-intensive HEIs. However, the current study does not directly equate its empirical validity with any single type of HEI, nor does it claim full generalization across HEI types. Applicability across different types of HEIs still requires further stratified validation. More broadly, because the scale and its criterion evidence were developed and tested specifically within the reverse-mobility framework, the extent to which perceived psychological exemption zone generalizes to other career-decision contexts beyond reverse mobility remains an open question; situating the construct within a broader career-development and organizational-psychology framework, beyond reverse mobility alone, is an important direction for future theoretical and empirical work. Exploratory factor analysis, parallel analysis, confirmatory factor analysis, reliability tests, convergent validity, and discriminant validity support the initial psychometric quality of PEZPS, while the criterion-related and incremental findings should be interpreted as preliminary evidence.

As highly educated talent face increasingly complex choices between market-oriented employment and HEI employment, it is important to understand how they evaluate work settings at the psychological level. PEZPS provides an empirical tool for explaining why doctoral talent shift from the “performance competition arena” toward HEIs perceived as “psychological exemption zones.” It also offers a new research foundation for HEI talent attraction, organizational health management in enterprises, and sustainable career development among doctoral talent.

## Data Availability

The original contributions presented in the study are included in the article/Supplementary material, further inquiries can be directed to the corresponding author.

## References

[ref1] Akkermans J. da Motta Veiga S. P. Hirschi A. Marciniak J. (2024). Career transitions across the lifespan: a review and research agenda. J. Vocat. Behav. 148:103957. doi: 10.1016/j.jvb.2023.103957, 42574925

[ref2] Allen M. S. Iliescu D. Greiff S. (2022). Single item measures in psychological science: a call to action. Eur. J. Psychol. Assess. 38, 1–5. doi: 10.1027/1015-5759/a000699

[ref3] Bakker A. B. Demerouti E. Sanz-Vergel A. I. (2023). Job demands-resources theory: ten years later. Annu. Rev. Organ. Psychol. Organ. Behav. 10, 25–53. doi: 10.1146/annurev-orgpsych-120920-053933

[ref4] Blustein D. L. Lysova E. I. Duffy R. D. (2023). Understanding decent work and meaningful work. Annu. Rev. Organ. Psychol. Organ. Behav. 10, 289–314. doi: 10.1146/annurev-orgpsych-031921-024847

[ref5] Boateng G. O. Neilands T. B. Frongillo E. A. Melgar-Quiñonez H. R. Young S. L. (2018). Best practices for developing and validating scales for health, social, and behavioral research: a primer. Front. Public Health 6:Article 149. doi: 10.3389/fpubh.2018.00149, 29942800 PMC6004510

[ref6] De Vos A. van der Heijden B. I. J. M. Akkermans J. (2020). Sustainable careers: towards a conceptual model. J. Vocat. Behav. 117:103196. doi: 10.1016/j.jvb.2018.06.011, 42574925

[ref7] Du P. Liu T. Xian X. (2026). From “performance competition arena” to “psychological exemption zone”: psychological safety mechanisms in reverse mobility. Front. Psychol. 17. doi: 10.3389/fpsyg.2026.1785539, 42382636 PMC13314783

[ref8] Edmondson A. C. (1999). Psychological safety and learning behavior in work teams. Adm. Sci. Q. 44, 350–383. doi: 10.2307/2666999

[ref9] Edmondson A. C. Bransby D. P. (2023). Psychological safety comes of age: observed themes in an established literature. Annu. Rev. Organ. Psychol. Organ. Behav. 10, 55–78. doi: 10.1146/annurev-orgpsych-120920-055217

[ref10] Edmondson A. C. Lei Z. (2014). Psychological safety: the history, renaissance, and future of an interpersonal construct. Annu. Rev. Organ. Psychol. Organ. Behav. 1, 23–43. doi: 10.1146/annurev-orgpsych-031413-091305

[ref11] Fisher G. G. Matthews R. A. Gibbons A. M. (2016). Developing and investigating the use of single-item measures in organizational research. J. Occup. Health Psychol. 21, 3–23. doi: 10.1037/a0039139, 25894198

[ref12] Fornell C. Larcker D. F. (1981). Evaluating structural equation models with unobservable variables and measurement error. J. Mark. Res. 18, 39–50. doi: 10.2307/3151312

[ref13] Gagné M. Parker S. L. Griffin M. A. Dunlop P. D. Knight C. Klonek F. E. . (2022). Understanding and shaping the future of work with self-determination theory. Nat. Rev. Psychol. 1, 378–392. doi: 10.1038/s44159-022-00056-w, 35574235 PMC9088153

[ref14] Goretzko D. Siemund K. Sterner P. (2024). Evaluating model fit of measurement models in confirmatory factor analysis. Educ. Psychol. Meas. 84, 123–144. doi: 10.1177/00131644231163813, 38250508 PMC10795573

[ref15] Groskurth K. Bluemke M. Lechner C. M. (2024). Why we need to abandon fixed cutoffs for goodness-of-fit indices: an extensive simulation and possible solutions. Behav. Res. Methods 56, 3891–3914. doi: 10.3758/s13428-023-02193-3, 37640961 PMC11133148

[ref16] Henseler J. Ringle C. M. Sarstedt M. (2015). A new criterion for assessing discriminant validity in variance-based structural equation modeling. J. Acad. Mark. Sci. 43, 115–135. doi: 10.1007/s11747-014-0403-8

[ref17] Hong M. Steedle J. T. Cheng Y. (2020). Methods of detecting insufficient effort responding: comparisons and practical recommendations. Educ. Psychol. Meas. 80, 312–345. doi: 10.1177/0013164419865316, 32158024 PMC7047258

[ref18] Horn J. L. (1965). A rationale and test for the number of factors in factor analysis. Psychometrika 30, 179–185. doi: 10.1007/BF02289447, 14306381

[ref19] Howard M. C. (2016). A review of exploratory factor analysis decisions and overview of current practices: what we are doing and how can we improve? Int. J. Hum.-Comput. Interact. 32, 51–62. doi: 10.1080/10447318.2015.1087664

[ref20] Hu L. T. Bentler P. M. (1999). Cutoff criteria for fit indexes in covariance structure analysis: conventional criteria versus new alternatives. Struct. Equ. Model. 6, 1–55. doi: 10.1080/10705519909540118

[ref21] Igolkina A. A. Meshcheryakov G. (2020). Semopy: a Python package for structural equation modeling. Struct. Equ. Model. 27, 952–963. doi: 10.1080/10705511.2019.1704289

[ref22] Kallio T. Siekkinen T. Pekkola E. Kivistö J. Nokkala T. Kuoppakangas P. (2024). Push and pull factors affecting in leaving academia. Tert. Educ. Manag. 30, 65–80. doi: 10.1007/s11233-024-09135-4

[ref23] Kossek E. E. Ruderman M. N. Braddy P. W. Hannum K. M. (2012). Work-nonwork boundary management profiles: a person-centered approach. J. Vocat. Behav. 81, 112–128. doi: 10.1016/j.jvb.2012.04.003

[ref24] Kreiner G. E. (2006). Consequences of work-home segmentation or integration: a person-environment fit perspective. J. Organ. Behav. 27, 485–507. doi: 10.1002/job.386

[ref25] Kurtessis J. N. Eisenberger R. Ford M. T. Buffardi L. C. Stewart K. A. Adis C. S. (2017). Perceived organizational support: a meta-analytic evaluation of organizational support theory. J. Manag. 43, 1854–1884. doi: 10.1177/0149206315575554

[ref26] Lawshe C. H. (1975). A quantitative approach to content validity. Pers. Psychol. 28, 563–575. doi: 10.1111/j.1744-6570.1975.tb01393.x

[ref27] Li H. Horta H. (2024). Perceived academic working conditions and career choices of Chinese PhD students. Stud. High. Educ. 49, 381–396. doi: 10.1080/03075079.2023.2264890

[ref28] Li H. Meng L. Xiong Y. Cook S. L. (2024). Job preferences and outcomes for China’s college graduates. China Q. 258, 529–547. doi: 10.1017/S0305741023001510

[ref29] Liu S. Wang H. Yu Z. (2025). Moving to campus: examining motivations and outcomes for Chinese journalists transitioning to higher education. Journalism, 27, 2511–2530. doi: 10.1177/14648849251365114 [published online 2025-07-31]

[ref30] McAnally K. Hagger M. S. (2024). Self-determination theory and workplace outcomes: a conceptual review and future research directions. Behav. Sci. 14:428. doi: 10.3390/bs14060428, 38920760 PMC11200516

[ref31] McNeish D. Wolf M. G. (2023). Dynamic fit index cutoffs for one-factor models. Behav. Res. Methods 55, 1157–1174. doi: 10.3758/s13428-022-01847-y, 35585278

[ref32] Mellner C. Peters P. Dragt M. J. Toivanen S. (2021). Predicting work-life conflict: types and levels of enacted and preferred work-nonwork boundary (in)congruence and perceived boundary control. Front. Psychol. 12:772537. doi: 10.3389/fpsyg.2021.772537, 34867680 PMC8636054

[ref33] Newman A. Donohue R. Eva N. (2017). Psychological safety: a systematic review of the literature. Hum. Resour. Manag. Rev. 27, 521–535. doi: 10.1016/j.hrmr.2017.01.001

[ref34] Podsakoff P. M. MacKenzie S. B. Lee J. Y. Podsakoff N. P. (2003). Common method biases in behavioral research: a critical review of the literature and recommended remedies. J. Appl. Psychol. 88, 879–903. doi: 10.1037/0021-9010.88.5.879, 14516251

[ref35] Rogers P. (2022). Best practices for your exploratory factor analysis: a factor tutorial. Rev. Adm. Contemp. 26:e210085. doi: 10.1590/1982-7849rac2022210085.en, 41099703

[ref36] Ryan R. M. Deci E. L. (2020). Intrinsic and extrinsic motivation from a self-determination theory perspective: definitions, theory, practices, and future directions. Contemp. Educ. Psychol. 61:101860. doi: 10.1016/j.cedpsych.2020.101860, 42574925

[ref37] Shoss M. K. (2017). Job insecurity: an integrative review and agenda for future research. J. Manag. 43, 1911–1939. doi: 10.1177/0149206317691574

[ref38] Sonnentag S. Fritz C. (2007). The recovery experience questionnaire: development and validation of a measure for assessing recuperation and unwinding from work. J. Occup. Health Psychol. 12, 204–221. doi: 10.1037/1076-8998.12.3.204, 17638488

[ref39] Sullivan S. E. Al Ariss A. (2021). Making sense of different perspectives on career transitions: a review and agenda for future research. Hum. Resour. Manag. Rev. 31:100727. doi: 10.1016/j.hrmr.2019.100727

[ref40] Sverke M. Låstad L. Hellgren J. Richter A. Näswall K. (2019). A meta-analysis of job insecurity and employee performance: testing temporal aspects, rating source, welfare regime, and union density as moderators. Int. J. Environ. Res. Public Health 16:2536. doi: 10.3390/ijerph16142536, 31315198 PMC6678210

[ref41] Tang L. Ruan N. Zheng G. (2025). Exploring Chinese doctoral graduates’ career choices and gender differences through the lens of rational choice theory. High. Educ. 90, 979–999. doi: 10.1007/s10734-024-01361-4

[ref42] van den Broeck A. Howard J. L. van Vaerenbergh Y. Leroy H. Gagné M. (2021). Beyond intrinsic and extrinsic motivation: a meta-analysis on self-determination theory’s multidimensional conceptualization of work motivation. Organ. Psychol. Rev. 11, 240–273. doi: 10.1177/20413866211006173

[ref43] Wang X. Wang W. L. (2024). The tenure track employment system in colleges and universities in China: a scoping review of the Chinese literature. Front. Psychol. 14:1271110. doi: 10.3389/fpsyg.2023.1271110 PMC10807458 38268816

[ref44] Ward M. K. Meade A. W. (2023). Dealing with careless responding in survey data: prevention, identification, and recommended best practices. Annu. Rev. Psychol. 74, 577–596. doi: 10.1146/annurev-psych-040422-045007, 35973734

[ref45] Wilson F. R. Pan W. Schumsky D. A. (2012). Recalculation of the critical values for Lawshe’s content validity ratio. Meas. Eval. Couns. Dev. 45, 197–210. doi: 10.1177/0748175612440286

[ref46] Worthington R. L. Whittaker T. A. (2006). Scale development research: a content analysis and recommendations for best practices. Counsel. Psychol. 34, 806–838. doi: 10.1177/0011000006288127

[ref47] Zapata-Ospina J. P. García-Valencia J. (2022). Validity based on content: a challenge in health measurement scales. J. Health Psychol. 27, 481–493. doi: 10.1177/1359105320953477, 32945184

[ref48] Zhai K. Moskal M. Read B. (2021). Compelled to compete: Chinese graduates on employment and social mobility after international and domestic study. Int. J. Educ. Dev. 84:Article 102432. doi: 10.1016/j.ijedudev.2021.102432

