# Supplementary material for: Development and initial psychometric validation of the psychological exemption zone perception scale in reverse mobility among doctoral talent
Source: Front Psychol. 2026 Oct 8;17:1923326. doi: 10.3389/fpsyg.2026.1923326 (PMC13687386; doi:10.3389/fpsyg.2026.1923326)
Supplement: Supplementary file 1 [file Table_1.DOCX]

**Supplementary Materials: Content Validity, Item Revision, and Additional Statistical Information for the Psychological Exemption Zone Perception Scale**

**Supplementary Appendix for PEZPS Scale Development and Initial Validation**

This supplementary material is an independent appendix to the main manuscript. It summarizes the expert content validity review, CVR-based screening, deleted items, revised retained items, final scale items, and additional statistical tables. To protect expert anonymity, only aggregated expert background information is reported, and no personally identifiable expert information is disclosed. Consistent with the main text, the items and statistical results in this appendix should be understood as preliminary validation materials in the context of reverse mobility among doctoral talent and evaluations of HEI/research work environments. Teaching-oriented, application-oriented, vocational undergraduate, and other non-research-intensive HEIs are important theoretical contexts of application, but their specific applicability and measurement invariance require further stratified validation.

# **Supplementary Table S1. Expert Background Information (Anonymous Aggregate Summary)**

Nine experts were invited to participate in the content validity review. All experts held a doctoral degree or senior professional title in organizational psychology, vocational psychology, human resource management, higher education research, or psychometrics. Their average length of relevant research or work experience was 13.5 years (range: 6-25 years). All experts completed ratings independently and provided open-ended written comments. To protect anonymity, individual expert identities are not disclosed.

The experts’ disciplinary backgrounds were distributed as follows:

| **Disciplinary field** | **Number of experts** |
| --- | --- |
| Organizational psychology | 2 |
| Vocational psychology | 2 |
| Human resource management | 2 |
| Higher education research | 2 |
| Psychometrics | 1 |

Note. Each expert was counted under one primary disciplinary field, yielding a total of nine experts. Some experts had multiple disciplinary backgrounds; the table reports the primary field self-identified by each expert.

# **Supplementary Table S2. Content Validity Review and CVR Results for Initial Items**

| **Item code** | **Theoretical dimension** | **Candidate item** | **ne** | **CVR** | **Decision** | **Expert comments/decision rationale** |
| --- | --- | --- | --- | --- | --- | --- |
| IS1 | Perceived institutional shelter | This work setting would allow me to feel relatively stable in my career development. | 9 | 1 | Retain | Core item; experts agreed that it represents perceived institutional stability. |
| IS2 | Perceived institutional shelter | The evaluation rules in this work setting are more stable than those in enterprises. | 8 | 0.78 | Retain after revision | Recommended for retention; some experts noted that “market-oriented organizations” should be clarified in the instructions. |
| IS3 | Perceived institutional shelter | This work setting would reduce my concerns about short-term performance fluctuations. | 9 | 1 | Retain | Core item; directly corresponds to buffering short-term performance risk. |
| IS4 | Perceived institutional shelter | This work setting would allow me to avoid facing high-risk competition. | 8 | 0.78 | Retain after revision | Retain after revision; experts suggested avoiding the implication of “no competition at all” and emphasizing exposure to high-risk competition. |
| IS5 | Perceived institutional shelter | This work setting would provide institutional protection for my long-term career development. | 9 | 1 | Retain | Core item; reflects institutional protection and long-term career development. |
| TS1 | Perceived time sovereignty | In this work setting, I could better organize my work rhythm. | 9 | 1 | Retain | Core item; clearly reflects control over work rhythm. |
| TS2 | Perceived time sovereignty | This work setting would give me more free time. | 8 | 0.78 | Retain after revision | Retain after revision; experts noted that “autonomous time” is not equivalent to leisure time. |
| TS3 | Perceived time sovereignty | This work setting would reduce work-related interruptions. | 8 | 0.78 | Retain after revision | Retain after revision; experts suggested emphasizing interruptions by immediate tasks rather than simply reduced workload. |
| TS4 | Perceived time sovereignty | In this work setting, I can make long-term plans. | 8 | 0.78 | Retain after revision | Retain after revision; experts considered the item relevant to long-term goal-based work planning and acceptable for this dimension. |
| TS5 | Perceived time sovereignty | This work setting would leave me time for thinking, learning, and self-development. | 9 | 1 | Retain | Core item; reflects time for thinking, learning, and self-development. |
| BC1 | Perceived boundary controllability | This work setting would help me maintain boundaries between work and life. | 9 | 1 | Retain | Core item; directly reflects work-life boundaries. |
| BC2 | Perceived boundary controllability | In this work setting, I can preserve private time. | 8 | 0.78 | Retain after revision | Retain after revision; experts suggested defining “private time” as non-work time. |
| BC3 | Perceived boundary controllability | This work setting would reduce the intrusion of work into life. | 8 | 0.78 | Retain after revision | Retain after revision; experts considered the item highly relevant to boundary intrusion. |
| BC4 | Perceived boundary controllability | This work setting would allow me to relax outside work. | 8 | 0.78 | Retain after revision | Retain after revision; experts suggested avoiding the implication that “HEI work is necessarily easy.” |
| BC5 | Perceived boundary controllability | This work setting would help me maintain a sense of order in life. | 9 | 1 | Retain | Core item; reflects a sense of order in life. |
| RS1 | Perceived restorative security | This work setting would help me recover from long-term occupational stress. | 9 | 1 | Retain | Core item; directly corresponds to restorability. |
| RS2 | Perceived restorative security | This work setting would allow me to restore my physical and psychological state. | 8 | 0.78 | Retain after revision | Retain after revision; experts suggested that wording about “stabilizing physical and psychological states” would be more moderate. |
| RS3 | Perceived restorative security | This work setting would reduce my concerns about occupational exhaustion. | 9 | 1 | Retain | Core item; reflects reduced risk of occupational exhaustion. |
| RS4 | Perceived restorative security | This work setting would allow me to avoid depleting my psychological energy. | 8 | 0.78 | Retain after revision | Retain after revision; experts suggested retaining “deplete psychological energy” while avoiding absolute wording. |
| RS5 | Perceived restorative security | This work setting would provide space for respite and readjustment. | 9 | 1 | Retain | Core item; reflects space for respite and readjustment. |
| MB1 | Perceived meaning-belonging | This work setting would support my pursuit of meaningful professional development. | 9 | 1 | Retain | Core item; represents meaningful professional development. |
| MB2 | Perceived meaning-belonging | In this work setting, I feel that my abilities are valuable. | 8 | 0.78 | Retain after revision | Retain after revision; experts suggested emphasizing the professional value of knowledge and abilities. |
| MB3 | Perceived meaning-belonging | This work setting would allow me to maintain an identity related to academia, teaching, or research. | 9 | 1 | Retain | Core item; reflects identity related to academia, teaching, or research. |
| MB4 | Perceived meaning-belonging | This work setting would make me a member of a community. | 8 | 0.78 | Retain after revision | Retain after revision; experts regarded a sense of community membership as key to the belonging dimension. |
| MB5 | Perceived meaning-belonging | This work setting would make me feel that my work is meaningful. | 8 | 0.78 | Retain after revision | Retain after revision; experts suggested distinguishing the item from general work-meaning items by emphasizing personal value realization. |
| DEL1 | Perceived institutional shelter | This work setting would guarantee stable income for me. | 5 | 0.11 | Delete | Delete. The item leans toward income security and may become a general career stability or salary-attractiveness item. |
| DEL2 | Perceived institutional shelter | This work setting would allow me to avoid competition altogether. | 3 | -0.33 | Delete | Delete. The wording is overly absolute; HEIs also involve competition, so it does not fit the real context. |
| DEL3 | Perceived time sovereignty | This work setting would allow me to avoid overtime completely. | 4 | -0.11 | Delete | Delete. The wording is overly absolute and may induce socially desirable responses. |
| DEL4 | Perceived restorative security | This work setting must be easier than enterprise work. | 2 | -0.56 | Delete | Delete. The comparison carries a value judgment, and the concept of “easy” is unclear. |
| DEL5 | Perceived meaning-belonging | This work setting would solve all my career anxiety. | 1 | -0.78 | Delete | Delete. The content is too broad to measure perceived psychological exemption zone precisely. |

Note. ne refers to the number of experts who rated an item as “highly relevant.” CVR = (ne - N/2)/(N/2), where N = 9. Decisions were determined by integrating CVR values and experts’ open-ended comments. The “candidate item” column presents the initial item wording used in expert review. For items retained after revision, revision trajectories and final wording are presented in Supplementary Tables S4 and S5, respectively.

Additional note. The “expert comments/decision rationale” column in Supplementary Table S2 summarizes the key points of experts’ open-ended comments. For items with ne = 8 (IS2, IS4, TS2, TS3, TS4, BC2, BC3, BC4, RS2, RS4, MB2, MB4, and MB5), at least one expert provided specific revision suggestions, most of which concerned avoiding absolute wording, clarifying dimensional boundaries, or strengthening contextualization. For items with ne = 9, although all experts endorsed the item, some still provided wording optimization suggestions; revision details are shown in Supplementary Table S4. All revisions were based on an integrated judgment of collective expert input and were not determined solely by CVR values.

# **Supplementary Table S3. Deleted Items and Reasons for Deletion**

| **Item code** | **Original theoretical dimension** | **Candidate item** | **CVR** | **Reason for deletion** |
| --- | --- | --- | --- | --- |
| DEL1 | Perceived institutional shelter | This work setting would guarantee stable income for me. | 0.11 | Leans toward income security and may become a general career stability or salary-attractiveness item. |
| DEL2 | Perceived institutional shelter | This work setting would allow me to avoid competition altogether. | -0.33 | The wording is overly absolute; HEIs also involve competition, so it does not fit the real context. |
| DEL3 | Perceived time sovereignty | This work setting would allow me to avoid overtime completely. | -0.11 | The wording is overly absolute and may induce socially desirable responses. |
| DEL4 | Perceived restorative security | This work setting must be easier than enterprise work. | -0.56 | The comparison carries a value judgment, and the concept of “easy” is unclear. |
| DEL5 | Perceived meaning-belonging | This work setting would solve all my career anxiety. | -0.78 | The content is too broad to measure perceived psychological exemption zone precisely. |

Note. Deleted items did not enter the formal survey version. Main reasons for deletion included CVR values below the recommended criterion, overly absolute wording, unclear construct boundaries, or confusion with non-core content such as income security and ease of work.

# **Supplementary Table S4. Revision Trajectories of Retained Items After Expert Review**

| **Item code** | **Theoretical dimension** | **Original item** | **Main expert comments** | **Revised item** | **Revision type** |
| --- | --- | --- | --- | --- | --- |
| IS2 | Perceived institutional shelter | The evaluation rules in this work setting are more stable than those in enterprises. | “Enterprise” was too narrow; compare with market-oriented organizations and emphasize predictability. | The evaluation rules in this work setting would be more predictable than those in market-oriented organizations. | Concept-boundary revision |
| IS4 | Perceived institutional shelter | This work setting would allow me to avoid facing high-risk competition. | “Not facing competition” was too absolute; revise to reduce exposure to long-term high-risk competition. | This work setting would make me feel that I do not have to remain in long-term, high-risk competition. | Revision of absolute wording |
| TS2 | Perceived time sovereignty | This work setting would give me more free time. | “Free time” could be misunderstood as leisure time; revise to relatively uninterrupted autonomous time. | This work setting would make me feel that I could have relatively uninterrupted autonomous time. | Concept-boundary revision |
| TS3 | Perceived time sovereignty | This work setting would reduce work-related interruptions. | “Work interruption” was too broad; emphasize immediate tasks and repeated interruptions. | This work setting would reduce my sense of being repeatedly interrupted by immediate tasks. | Contextualized revision |
| TS4 | Perceived time sovereignty | In this work setting, I can make long-term plans. | Clarify that the item concerns planning work around long-term goals, rather than general life planning. | In this work setting, I would be more likely to plan my work around long-term goals. | Referent specification revision |
| BC2 | Perceived boundary controllability | In this work setting, I can preserve private time. | Weaken absolute wording by using “more likely” and emphasize time that belongs to oneself. | In this work setting, I would be more likely to preserve personal time for myself. | Revision of absolute wording |
| BC3 | Perceived boundary controllability | This work setting would reduce the intrusion of work into life. | Distinguish family life from personal life and emphasize continuous intrusion. | This work setting would reduce the continuous intrusion of work into family or personal life. | Content specification |
| BC4 | Perceived boundary controllability | This work setting would allow me to relax outside work. | Experts noted that the item could imply that HEI work is necessarily easy; revise to emphasize subjective experience and genuine relaxation. | This work setting would make me feel that I could truly relax outside work. | Subjectivizing revision |
| RS2 | Perceived restorative security | This work setting would allow me to restore my physical and psychological state. | The wording was somewhat direct; revise to “physical and psychological state gradually stabilizes.” | This work setting would make me feel that my physical and psychological state could gradually stabilize. | Moderation of tone |
| RS4 | Perceived restorative security | This work setting would allow me to avoid depleting my psychological energy. | “Do not have to” was too absolute; revise to “do not have to continuously deplete.” | This work setting would make me feel that I do not have to continuously deplete my psychological energy. | Revision of absolute wording |
| MB2 | Perceived meaning-belonging | In this work setting, I feel that my abilities are valuable. | Add knowledge and abilities to emphasize doctoral talent’s professional capital. | In this work setting, I would be more likely to feel that my knowledge and abilities are valuable. | Referent specification |
| MB4 | Perceived meaning-belonging | This work setting would make me a member of a community. | Emphasize a long-term professional community rather than general belonging. | This work setting would make me feel that I could become a member of a long-term professional community. | Conceptual precision |
| MB5 | Perceived meaning-belonging | This work setting would make me feel that my work is meaningful. | Too close to general work-meaning items; emphasize personal value realization beyond task completion. | This work setting would make me feel that work is not only about completing tasks but also about realizing personal value. | Differentiation from adjacent constructs |

Note. This table presents only the items retained after revision based on expert comments. It documents the specific basis for semantic revision, construct-boundary revision, and differentiation from adjacent dimensions.

# **Supplementary Table S5. Formal PEZPS Items**

| **Item code** | **Dimension** | **Original item (English translation)** | **Recommended English wording** | **Scoring** |
| --- | --- | --- | --- | --- |
| IS1 | Perceived institutional shelter | This work setting would allow me to feel relatively stable in my career development. | This work setting would allow me to feel relatively stable in my career development. | 1 = strongly disagree; 5 = strongly agree |
| IS2 | Perceived institutional shelter | The evaluation rules in this work setting would be more predictable than those in market-oriented organizations. | The evaluation rules in this work setting would be more predictable than those in market-oriented organizations. | 1 = strongly disagree; 5 = strongly agree |
| IS3 | Perceived institutional shelter | This work setting would reduce my concerns about short-term performance fluctuations. | This work setting would reduce my concerns about short-term performance fluctuations. | 1 = strongly disagree; 5 = strongly agree |
| IS4 | Perceived institutional shelter | This work setting would make me feel that I do not have to remain in long-term, high-risk competition. | This work setting would make me feel that I do not have to remain in long-term, high-risk competition. | 1 = strongly disagree; 5 = strongly agree |
| IS5 | Perceived institutional shelter | This work setting would provide institutional protection for my long-term career development. | This work setting would provide institutional protection for my long-term career development. | 1 = strongly disagree; 5 = strongly agree |
| TS1 | Perceived time sovereignty | In this work setting, I could better organize my work rhythm. | In this work setting, I could better organize my work rhythm. | 1 = strongly disagree; 5 = strongly agree |
| TS2 | Perceived time sovereignty | This work setting would make me feel that I could have relatively uninterrupted autonomous time. | This work setting would make me feel that I could have relatively uninterrupted autonomous time. | 1 = strongly disagree; 5 = strongly agree |
| TS3 | Perceived time sovereignty | This work setting would reduce my sense of being repeatedly interrupted by immediate tasks. | This work setting would reduce my sense of being repeatedly interrupted by immediate tasks. | 1 = strongly disagree; 5 = strongly agree |
| TS4 | Perceived time sovereignty | In this work setting, I would be more likely to plan my work around long-term goals. | In this work setting, I would be more likely to plan my work around long-term goals. | 1 = strongly disagree; 5 = strongly agree |
| TS5 | Perceived time sovereignty | This work setting would leave me time for thinking, learning, and self-development. | This work setting would leave me time for thinking, learning, and self-development. | 1 = strongly disagree; 5 = strongly agree |
| BC1 | Perceived boundary controllability | This work setting would help me maintain boundaries between work and life. | This work setting would help me maintain boundaries between work and life. | 1 = strongly disagree; 5 = strongly agree |
| BC2 | Perceived boundary controllability | In this work setting, I would be more likely to preserve personal time for myself. | In this work setting, I would be more likely to preserve personal time for myself. | 1 = strongly disagree; 5 = strongly agree |
| BC3 | Perceived boundary controllability | This work setting would reduce the continuous intrusion of work into family or personal life. | This work setting would reduce the continuous intrusion of work into family or personal life. | 1 = strongly disagree; 5 = strongly agree |
| BC4 | Perceived boundary controllability | This work setting would make me feel that I could truly relax outside work. | This work setting would make me feel that I could truly relax outside work. | 1 = strongly disagree; 5 = strongly agree |
| BC5 | Perceived boundary controllability | This work setting would help me maintain a sense of order in life. | This work setting would help me maintain a sense of order in life. | 1 = strongly disagree; 5 = strongly agree |
| RS1 | Perceived restorative security | This work setting would help me recover from long-term occupational stress. | This work setting would help me recover from long-term occupational stress. | 1 = strongly disagree; 5 = strongly agree |
| RS2 | Perceived restorative security | This work setting would make me feel that my physical and psychological state could gradually stabilize. | This work setting would make me feel that my physical and psychological state could gradually stabilize. | 1 = strongly disagree; 5 = strongly agree |
| RS3 | Perceived restorative security | This work setting would reduce my concerns about occupational exhaustion. | This work setting would reduce my concerns about occupational exhaustion. | 1 = strongly disagree; 5 = strongly agree |
| RS4 | Perceived restorative security | This work setting would make me feel that I do not have to continuously deplete my psychological energy. | This work setting would make me feel that I do not have to continuously deplete my psychological energy. | 1 = strongly disagree; 5 = strongly agree |
| RS5 | Perceived restorative security | This work setting would provide space for respite and readjustment. | This work setting would provide space for respite and readjustment. | 1 = strongly disagree; 5 = strongly agree |
| MB1 | Perceived meaning-belonging | This work setting would support my pursuit of meaningful professional development. | This work setting would support my pursuit of meaningful professional development. | 1 = strongly disagree; 5 = strongly agree |
| MB2 | Perceived meaning-belonging | In this work setting, I would be more likely to feel that my knowledge and abilities are valuable. | In this work setting, I would be more likely to feel that my knowledge and abilities are valuable. | 1 = strongly disagree; 5 = strongly agree |
| MB3 | Perceived meaning-belonging | This work setting would allow me to maintain an identity related to academia, teaching, or research. | This work setting would allow me to maintain an identity related to academia, teaching, or research. | 1 = strongly disagree; 5 = strongly agree |
| MB4 | Perceived meaning-belonging | This work setting would make me feel that I could become a member of a long-term professional community. | This work setting would make me feel that I could become a member of a long-term professional community. | 1 = strongly disagree; 5 = strongly agree |
| MB5 | Perceived meaning-belonging | This work setting would make me feel that work is not only about completing tasks but also about realizing personal value. | This work setting would make me feel that work is not only about completing tasks but also about realizing personal value. | 1 = strongly disagree; 5 = strongly agree |

Note. All items are scored on a 5-point Likert scale, where 1 = strongly disagree and 5 = strongly agree. The total scale score may be calculated as the mean of all 25 items, and dimension scores may be calculated as the mean of the five items within each dimension. In administration, “this work setting” may be replaced with a specific target organizational type. In the present formal survey, “this work setting” referred to the HEI/research work environment that respondents were considering, had entered, or were comparing; respondents were not required to use any specific type of HEI as the only referent. In light of prior qualitative research, teaching-oriented general undergraduate institutions, application-oriented undergraduate institutions, vocational undergraduate institutions, and other non-research-intensive HEIs may serve as important theoretical contexts for PEZPS. However, high-intensity “up-or-out” positions in high-level research universities or positions with very high short-cycle research performance pressure should not be directly equated with a “psychological exemption zone.” Nor should the empirical validity of the scale be interpreted as applying only to teaching-oriented or non-research-intensive HEIs. Applicability and measurement invariance across different HEI types are issues for subsequent stratified validation and use-scope clarification, and do not undermine the basic evidence of initial scale validation in the context of reverse mobility among doctoral talent and evaluation of HEI/research work settings.

# **Supplementary Table S6. Summary of Exploratory Factor Analysis Item Loadings**

| **Item** | **Theoretical dimension** | **Primary loading** | **Maximum cross-loading** | **Communality** |
| --- | --- | --- | --- | --- |
| IS1 | Perceived institutional shelter | 0.737 | 0.056 | 0.549 |
| IS2 | Perceived institutional shelter | 0.778 | 0.068 | 0.610 |
| IS3 | Perceived institutional shelter | 0.821 | 0.072 | 0.685 |
| IS4 | Perceived institutional shelter | 0.799 | 0.109 | 0.659 |
| IS5 | Perceived institutional shelter | 0.846 | 0.039 | 0.719 |
| TS1 | Perceived time sovereignty | 0.789 | 0.053 | 0.626 |
| TS2 | Perceived time sovereignty | 0.788 | 0.036 | 0.624 |
| TS3 | Perceived time sovereignty | 0.821 | 0.079 | 0.682 |
| TS4 | Perceived time sovereignty | 0.825 | 0.038 | 0.682 |
| TS5 | Perceived time sovereignty | 0.862 | 0.045 | 0.747 |
| BC1 | Perceived boundary controllability | 0.768 | 0.142 | 0.624 |
| BC2 | Perceived boundary controllability | 0.773 | 0.086 | 0.613 |
| BC3 | Perceived boundary controllability | 0.895 | 0.093 | 0.820 |
| BC4 | Perceived boundary controllability | 0.800 | 0.136 | 0.667 |
| BC5 | Perceived boundary controllability | 0.791 | 0.108 | 0.647 |
| RS1 | Perceived restorative security | 0.790 | 0.031 | 0.625 |
| RS2 | Perceived restorative security | 0.804 | 0.046 | 0.649 |
| RS3 | Perceived restorative security | 0.865 | 0.099 | 0.761 |
| RS4 | Perceived restorative security | 0.759 | 0.060 | 0.582 |
| RS5 | Perceived restorative security | 0.859 | 0.045 | 0.741 |
| MB1 | Perceived meaning-belonging | 0.792 | 0.060 | 0.636 |
| MB2 | Perceived meaning-belonging | 0.753 | 0.054 | 0.576 |
| MB3 | Perceived meaning-belonging | 0.759 | 0.076 | 0.584 |
| MB4 | Perceived meaning-belonging | 0.853 | 0.092 | 0.737 |
| MB5 | Perceived meaning-belonging | 0.776 | 0.113 | 0.616 |

Note. Supplementary Table S6 reports a summary of rotated five-factor loadings in the exploratory sample (n = 335). Principal axis factoring was used for extraction, and Promax oblique rotation (Kappa = 4) was used for rotation. Primary loadings were taken from the rotated pattern matrix and are reported to three decimals. The primary loading refers to the item’s pattern loading on its assigned theoretical dimension; the maximum cross-loading refers to the largest absolute pattern loading on any non-assigned dimension; and communality refers to the corresponding item communality in the five-factor solution. Because statistical software packages may differ slightly in implementations of oblique rotation and loading standardization, the item-level loadings in this table are based on the output of the same Python analysis script and parameter settings used in this study (factor_analyzer, principal axis extraction + Promax rotation, fixed random seed). The full script may be obtained from the corresponding author upon reasonable request for research purposes, subject to appropriate ethical and data-use considerations.

# **Supplementary Table S7. Standardized Factor Loadings from Confirmatory Factor Analysis**

| **Item** | **Dimension** | **Standardized loading λ** |
| --- | --- | --- |
| IS1 | Perceived institutional shelter | 0.780 |
| IS2 | Perceived institutional shelter | 0.728 |
| IS3 | Perceived institutional shelter | 0.768 |
| IS4 | Perceived institutional shelter | 0.693 |
| IS5 | Perceived institutional shelter | 0.791 |
| TS1 | Perceived time sovereignty | 0.762 |
| TS2 | Perceived time sovereignty | 0.769 |
| TS3 | Perceived time sovereignty | 0.812 |
| TS4 | Perceived time sovereignty | 0.720 |
| TS5 | Perceived time sovereignty | 0.797 |
| BC1 | Perceived boundary controllability | 0.719 |
| BC2 | Perceived boundary controllability | 0.762 |
| BC3 | Perceived boundary controllability | 0.825 |
| BC4 | Perceived boundary controllability | 0.742 |
| BC5 | Perceived boundary controllability | 0.727 |
| RS1 | Perceived restorative security | 0.716 |
| RS2 | Perceived restorative security | 0.738 |
| RS3 | Perceived restorative security | 0.770 |
| RS4 | Perceived restorative security | 0.729 |
| RS5 | Perceived restorative security | 0.774 |
| MB1 | Perceived meaning-belonging | 0.690 |
| MB2 | Perceived meaning-belonging | 0.724 |
| MB3 | Perceived meaning-belonging | 0.655 |
| MB4 | Perceived meaning-belonging | 0.762 |
| MB5 | Perceived meaning-belonging | 0.706 |

Note. N = 503 (validation sample). λ denotes standardized factor loading; all loadings were significant at p < .001.

# **Supplementary Table S8. HTMT Test of Discriminant Validity**

| **Dimension 1** | **Dimension 2** | **HTMT** |
| --- | --- | --- |
| Perceived institutional shelter | Perceived time sovereignty | 0.305 |
| Perceived institutional shelter | Perceived boundary controllability | 0.271 |
| Perceived institutional shelter | Perceived restorative security | 0.572 |
| Perceived institutional shelter | Perceived meaning-belonging | 0.325 |
| Perceived time sovereignty | Perceived boundary controllability | 0.700 |
| Perceived time sovereignty | Perceived restorative security | 0.299 |
| Perceived time sovereignty | Perceived meaning-belonging | 0.240 |
| Perceived boundary controllability | Perceived restorative security | 0.253 |
| Perceived boundary controllability | Perceived meaning-belonging | 0.253 |
| Perceived restorative security | Perceived meaning-belonging | 0.413 |

Note. All HTMT values were below .85, supporting acceptable discriminant validity among dimensions.

# **Supplementary Table S9. Fornell-Larcker Test of Discriminant Validity**

| **Dimension** | **Institutional shelter** | **Time sovereignty** | **Boundary controllability** | **Restorative security** | **Meaning-belonging** |
| --- | --- | --- | --- | --- | --- |
| Perceived institutional shelter | 0.753 |  |  |  |  |
| Perceived time sovereignty | 0.308 | 0.773 |  |  |  |
| Perceived boundary controllability | 0.264 | 0.697 | 0.756 |  |  |
| Perceived restorative security | 0.566 | 0.301 | 0.253 | 0.746 |  |
| Perceived meaning-belonging | 0.319 | 0.240 | 0.257 | 0.412 | 0.709 |

Note. The diagonal values are the square roots of AVE for each dimension, and the lower triangle reports inter-dimensional correlations. For each dimension, the square root of AVE exceeded its correlations with other dimensions, supporting discriminant validity.

# **Supplementary Table S10. PEZPS Means Across Doctoral Talent Groups**

| **Group** | **n** | **Institutional shelter** | **Time sovereignty** | **Boundary controllability** | **Restorative security** | **Meaning-belonging** | **Total score** |
| --- | --- | --- | --- | --- | --- | --- | --- |
| Senior doctoral students | 304 | 3.05 | 3.02 | 3.04 | 3.02 | 3.03 | 3.03 |
| Doctoral graduates within the past five years | 158 | 3.09 | 3.05 | 3.00 | 3.09 | 3.10 | 3.07 |
| Doctoral degree holders working in enterprises | 121 | 3.04 | 3.17 | 3.28 | 3.04 | 2.99 | 3.11 |
| Individuals who moved from enterprises to HEIs | 104 | 3.29 | 3.24 | 3.32 | 3.31 | 3.21 | 3.27 |
| Postdoctoral researchers/early-career HEI faculty | 151 | 3.17 | 2.92 | 3.03 | 3.11 | 3.25 | 3.09 |

Note. Postdoctoral researchers and early-career HEI faculty were combined in this study because both are at early stages of academic careers and some early-career faculty transition from postdoctoral positions. However, differences in job nature and career stability remain, and results should be interpreted with caution. This table is descriptive only and is not intended for strong inferential interpretation of cross-group mean differences.

# **Supplementary Table S11. Five-Factor Model Fit Indices Across Career-Stage Groups**

| **Group** | **n** | **χ²/df** | **CFI** | **TLI** | **RMSEA** |
| --- | --- | --- | --- | --- | --- |
| Senior doctoral students | 304 | 1.75 | 0.947 | 0.940 | 0.050 |
| Doctoral graduates within the past five years | 158 | 1.59 | 0.928 | 0.919 | 0.061 |
| Postdoctoral researchers/early-career HEI faculty | 151 | 1.41 | 0.929 | 0.920 | 0.052 |
| Doctoral degree holders working in enterprises | 121 | 1.42 | 0.931 | 0.922 | 0.060 |
| Individuals who moved from enterprises to HEIs | 104 | 1.35 | 0.936 | 0.927 | 0.058 |

Note. A correlated first-order five-factor model was fitted separately for each group using maximum likelihood (ML) estimation. The table reports χ²/df, CFI, TLI, and RMSEA. Because of limited sample sizes in some groups, these results provide only preliminary evidence for configural consistency of the five-factor structure across career-stage groups and are not equivalent to multi-group measurement invariance tests of loading or intercept invariance.

# **Supplementary Table S12. Criterion Variable Item Wording**

| **Variable** | **Item wording (English)** | **Scoring method** | **Theoretical use** |
| --- | --- | --- | --- |
| psychological safety need | In my career choice, I place great importance on the stability and psychological safety of the work environment. | 1-5 points | Convergent validity: testing the positive association between PEZPS and safety need |
| occupational burnout | I often feel physically and mentally exhausted because of work. | 1-5 points | Stress-driven criterion: testing the positive association between PEZPS and exhaustion |
| job insecurity | I worry that I may lose my current job in the future. | 1-5 points | Stress-driven criterion: testing the positive association between PEZPS and uncertainty concerns |
| meaningfulness of work | I believe that my work enables me to realize personal value. | 1-5 points | Convergent validity: testing the positive association between PEZPS and meaning seeking |
| boundary-control need | I hope to have clear personal time boundaries at work. | 1-5 points | Convergent validity: testing the positive association between PEZPS and boundary needs |
| reverse mobility intention | I tend to leave an enterprise and work in a higher education institution or research institute. | 1-5 points | Primary criterion outcome: testing the predictive power of PEZPS for mobility intention |
| HEI job-search intention | I plan to apply for faculty or research positions in higher education institutions within the next year. | 1-5 points | Criterion validity: testing the positive association between PEZPS and HEI job-search willingness |
| enterprise retention intention | I intend to continue working in my current enterprise or market-oriented organization. | 1-5 points | Criterion validity: testing the negative association between PEZPS and enterprise retention intention |

Note. All criterion variables used in this study were brief single-item indicators, intended to control questionnaire length while providing preliminary tests of the criterion-related and incremental validity of PEZPS. Each variable was rated on a 5-point Likert scale (1 = strongly disagree, 5 = strongly agree). Cronbach’s α was not calculated for single-item indicators.
